# Bio-inspired lotus-fiber and mussel-based multifunctional hydrogels for wound healing: super-stretchability, self-healing, adhesion and antibacterial properties

**DOI:** 10.1093/rb/rbaf031

**Published:** 2025-04-26

**Authors:** Xiaoling Yang, Chenchen Li, Bo Li, Yuanyuan Zhang, Jinping Li, Na Liu, Xin Nie, Dawei Zhang, Ming Zhou, Xiaoling Liao

**Affiliations:** School of New Energy and Material, Southwest Petroleum University, Chengdu 610500, China; Chongqing Engineering Laboratory of Nano/Micro Biomedical Detection Technology, Chongqing University of Science and Technology, Chongqing 401331, China; Chongqing Engineering Laboratory of Nano/Micro Biomedical Detection Technology, Chongqing University of Science and Technology, Chongqing 401331, China; Chongqing Engineering Laboratory of Nano/Micro Biomedical Detection Technology, Chongqing University of Science and Technology, Chongqing 401331, China; Chongqing Engineering Laboratory of Nano/Micro Biomedical Detection Technology, Chongqing University of Science and Technology, Chongqing 401331, China; Chongqing Engineering Laboratory of Nano/Micro Biomedical Detection Technology, Chongqing University of Science and Technology, Chongqing 401331, China; Chongqing Engineering Laboratory of Nano/Micro Biomedical Detection Technology, Chongqing University of Science and Technology, Chongqing 401331, China; Chongqing Engineering Laboratory of Nano/Micro Biomedical Detection Technology, Chongqing University of Science and Technology, Chongqing 401331, China; Department of Orthopedics, The 960th Hospital of the PLA Joint Logistice Support Force, Jinan 250031, China; School of New Energy and Material, Southwest Petroleum University, Chengdu 610500, China; Chongqing Engineering Laboratory of Nano/Micro Biomedical Detection Technology, Chongqing University of Science and Technology, Chongqing 401331, China

**Keywords:** wound dressing, hydrogel, multifunctional, bacterial cellulose, lotus-fiber

## Abstract

Hydrogel-based wound dressings, which facilitate rapid wound closure and healing, are essential for effective wound management. However, the development of an ideal hydrogel that possesses excellent mechanical properties, effective self-healing capabilities, tissue adherence and antimicrobial characteristics for wound dressing presents a significant challenge in clinical settings. Inspired by lotus-fiber and mussel, we synthesized a novel multifunctional hydrogel composed of bacterial cellulose-reinforced dopamine-grafted oxidized hyaluronic acid/polyacrylamide (OHA-DA/PAM/BC). This was achieved through a one-pot reaction that employed free radical polymerization of acrylamide, dynamic Schiff bonding and intermolecular hydrogen bonding. Compared with the pure PAM hydrogels, which exhibited an elongation at break of 4022% and a maximum tensile strength of 26.42 kPa, the OHA-DA/PAM hydrogel demonstrated significantly enhanced stretchability at 9949% and an increased tensile strength of 34.73 kPa when 0.3% OHA-DA was incorporated during hydrogel formulation. Notably, the addition of 0.8% BC significantly enhanced the tensile strength to 57.04 kPa and super-stretchability to 10679%. The OHA-DA/PAM/BC hydrogel also exhibited remarkable self-healing capabilities, achieving a mechanical recovery of 84.74% within 12 h. Additionally, its adhesive and injectable properties are advantageous for dynamic wound repair. Furthermore, the OHA-DA/PAM/BC hydrogel exhibited minimal hemolytic activity and potent intrinsic antibacterial properties against both *Escherichia coli* and *Staphylococcus aureus*. In a mouse model of wound healing, this hydrogel reduced the healing duration to 14 days while enhancing the regeneration of both skin structure and function. Histological analyses further revealed that the hydrogel significantly promoted the development of well-organized granulation tissue, angiogenic tissue and collagen accumulation in the wound region. This study successfully developed an OHA-DA/PAM/BC multifunctional hydrogel characterized by exceptional stretchability, self-healing, adhesiveness, injectability and antibacterial activity, demonstrating a significant impact on wound healing *in vivo*. These findings indicated that the OHA-DA/PAM/BC hydrogel holds substantial potential as wound dressings for future clinical applications.

## Graphical abstract

**Figure:**
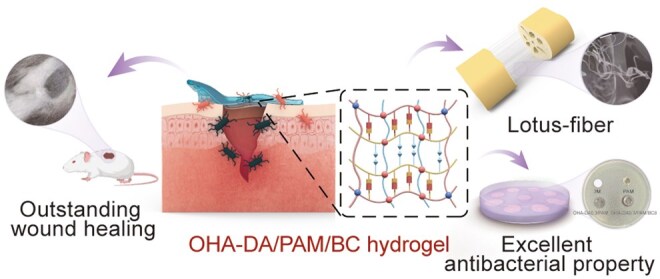

## Introduction

The skin serves as a vital protective barrier for the human body, fulfilling essential functions such as regulating body temperature, maintaining electrolyte and fluid balance, and facilitating sensory perception [1–4]. However, human skin is highly susceptible to injury, which can result in wounds and increase the risk of bacterial infections. These infections often cause severe inflammation and hinder the healing process, potentially resulting in serious consequences [5]. As a result, the quality of life for affected patients may be significantly diminished. Wound healing is recognized as one of the most complex and dynamic biological processes in the human body [6], making it challenging for adults with skin injuries to achieve full restoration of skin function, as observed in infants. Therefore, it is imperative to develop wound dressings that possess antibacterial, anti-inflammatory, hemostatic and wound healing properties [7]. Over the past few decades, various biomaterials have been developed for use as wound dressings, including films [8], foams [9], hydrogels [10, 11] and hydrocolloids [12]. Hydrogels are mainly employed in wound dressings due to their significant swelling properties, hydrophilicity, high oxygen permeability, three-dimensional crosslinked polymer network, exceptional biocompatibility and resemblance to the extracellular matrix (ECM) [13–15]. However, conventional hydrogel dressings face several limitations, including inadequate mechanical strength, poor tissue adhesion, lack of self-healing and degradability features, insufficient antibacterial effectiveness and inadaptability to wound repair [16, 17], which restricts their clinical application. Hence, there is an urgent need for the advancement of innovative multifunctional hydrogel wound dressings.

Recently, various polymers have been employed to develop a range of hydrogels, which are typically categorized as either natural or synthetic polymers. Hydrogels derived from natural polymers such as chitosan [18–20], hyaluronic acid (HA) [21, 22], sodium alginate [23, 24], gelatin [25, 26] and silk protein [27–29] generally exhibit properties that are consistent with biological tissues due to their similar compositions. HA, a disaccharide classified as a non-sulfated glycosaminoglycan (GAG), consists of two essential components: D-glucuronic acid and N-acetylglucosamine. These components are crucial as primary elements of the ECM within the skin. Hydrogels based on HA have been extensively studied for use in wound dressings due to their inherently derived, non-immunogenic, biodegradable and non-adhesive properties, as well as their ability to suppress bacterial proliferation, reduce inflammation and facilitate wound healing [30]. However, the mechanical limitations of unmodified HA hydrogels restrict their effectiveness in wound dressings. In comparison to natural polymers, synthetic polymers exhibit superior hydrophobic properties and mechanical strength. Consequently, synthetic polymers such as poly(vinyl alcohol) (PVA) [31], polyacrylamide (PAM) [32] and polyacrylic acid (PAA) [33] emerge as promising candidates for wound dressing applications. Among these, PAM hydrogels are synthesized from acrylamide (AM), forming a three-dimensional network that retains a significant volume of water and mimics the physical characteristics and chemical composition of the ECM. These hydrogels have been specifically developed for biomedical applications, including trauma treatment. The integration of PAM into HA hydrogels has the potential to enhance the mechanical strength of the natural polymers. However, the inadequate mechanical properties of hydrogels continue to limit their use in wound dressings. Drawing inspiration from the natural lotus-fiber, a widely cultivated aquatic perennial plant [34], bacterial cellulose (BC) can be incorporated into the composite to promote energy dissipation. An ancient Chinese proverb states, ‘while the lotus roots might break, the fibers stay connected’, highlighting the unique structure and mechanical properties of the fibers [35]. Therefore, the addition of BC fibers can replicate the distinctive structure of lotus fibers, thereby enhancing the mechanical properties of the hydrogels.

The adhesion of hydrogels is crucial for the effective fixation of wound dressings. However, the limited adhesive properties of traditional hydrogels restrict their applicability in this context. Drawing inspiration from the chemical composition of natural mussels, which exhibit strong adhesion to various material surfaces, researchers have incorporated catechol groups into hydrogels to enhance their adhesive properties [36, 37]. Dopamine (DA), a derivative of tyrosine present in mussel proteins, is frequently employed to improve the adhesion of hydrogels. Nevertheless, in many previous studies, DA was typically self-polymerized under alkaline conditions to form black polydopamine (PDA), which, while providing adhesion to the hydrogel, adversely affects its transparency—an undesirable trait for monitoring wound recovery. Moreover, an optimal hydrogel for wound dressings should possess additional functionalities, including effective self-healing capabilities to protect wounds from further injury and antibacterial properties to prevent infections. The chemical grafting of DA onto the hydrogel backbone preserves a significant number of catechol moieties, thereby potentially enhancing the tissue adhesion and antibacterial characteristics of the composite hydrogels.

**Figure 1.**
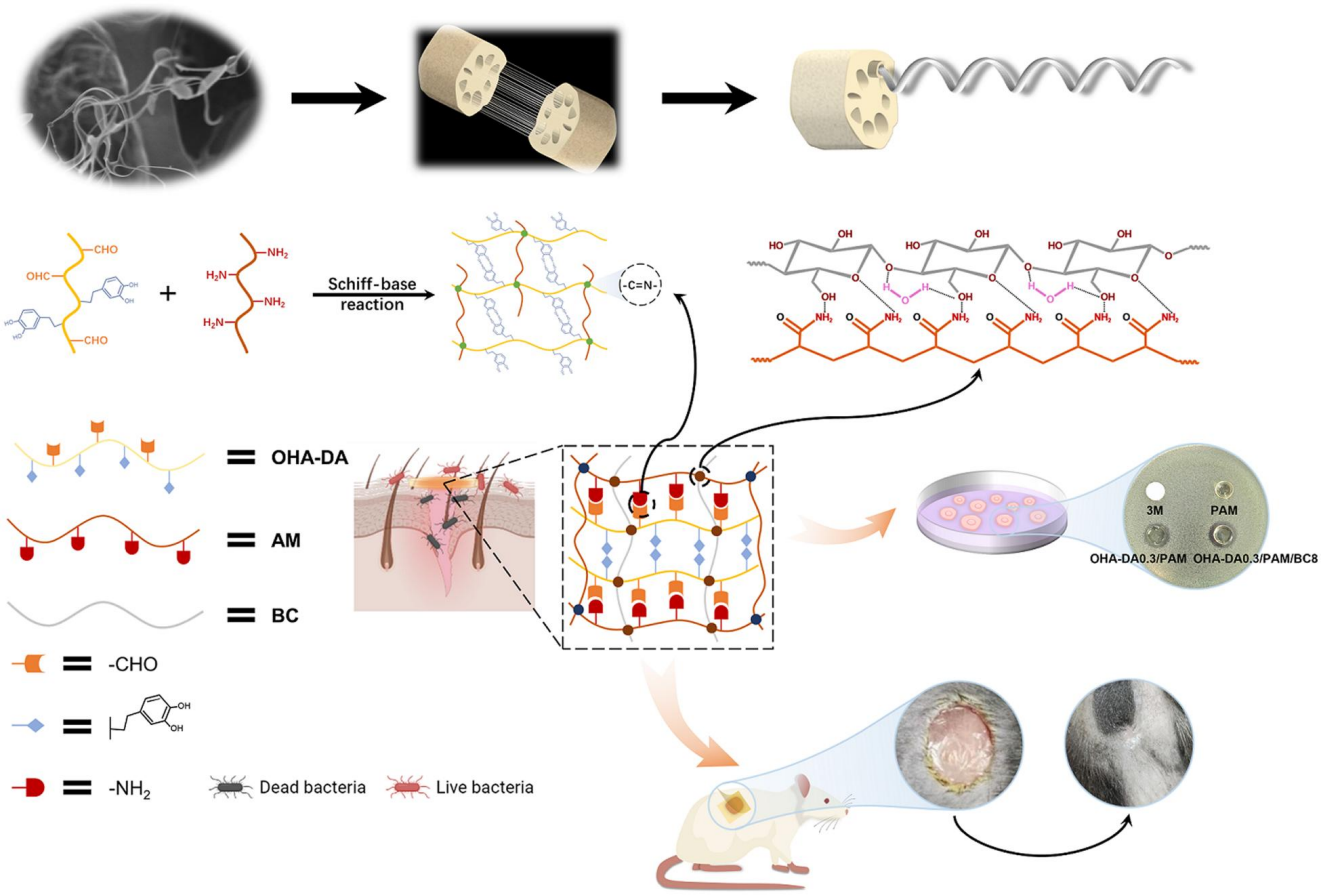
Diagram illustrating the preparation process of the OHA-DA/PAM/BC hydrogel, a visual representation of the antibacterial mechanism and its potential uses in wound dressing (created with BioRender.com).

In this study, we fabricated a DA-grafted oxidized HA/PAM (OHA-DA/PAM) hydrogel incorporating BC, drawing inspiration from the lotus-fiber and mussel. We investigated the effects of varying OHA-DA and BC contents on the hydrogels, with a focus on their microscopic structure, mechanical properties, self-healing properties, swelling behavior and adhesion capabilities. Furthermore, we examined the hemolytic activity and impact of these materials on both gram-positive and gram-negative bacteria. To evaluate the effectiveness of the hydrogel dressings in promoting wound healing, we established a mouse wound model (Figure 1).

## Materials and methods

### Materials

HA, dopamine hydrochloride (DA), sodium periodate (NaIO_4_), ethylene glycol (EG), AM, ammonium persulfate (APS), N,N′-methylenebisacrylamide (BIS), N,N,N′,N′-tetramethyl ethylenediamine (TEMED), 1-ethyl-3-(3-dimethylaminopropyl)carbodiimide (EDC) and N-hydroxy succinimide (NHS) were obtained from Aladdin Co., Ltd (Shanghai, China). Ultrapure (UP) water was employed for all experimental procedures, while all other chemicals were of analytical grade and were used as received.

BC membranes were sourced from Hainan Yide Food Industry Co., Ltd (China). After cutting the BC into small pieces, it was purified by immersion in a 0.1 M NaOH solution at 80°C for 4 h, followed by rinsing to achieve a neutral pH with UP water. The prepared BC was then redispersed in a specific volume of deionized water and homogenized for 30 min using a homogenizer (30 000 r/min) to obtain a homogeneous aqueous dispersion with mass concentrations of 0.3%, 0.5%, 0.8%, 1% and 2% [38].

### Synthesis and characterization of OHA-DA

OHA was prepared following a modified approach based on previous research [39–42]. Briefly, 2 g of HA was mixed with 200 ml of deionized water (DI) while stirring vigorously. A solution of 3.17 g of NaIO_4_ in 20 ml of deionized water was then added slowly to the mixture, which was kept in the dark for 8 h at 25°C. To terminate the reaction, 1 ml of ethylene glycol was added, and the mixture was stirred for an additional hour. Subsequently, the solution underwent dialysis (MWCO 3500) with deionized water over three days. The product was then lyophilized for further use.

To enhance the adhesive properties of the hydrogel, DA was grafted onto the OHA backbone to prepare DA-grafted OHA (OHA-DA) utilizing the classic EDC/NHS coupling reaction [16]. Initially, OHA (1 g) was completely dissolved in 100 ml of PBS (50 mmol/L, pH = 5.5) under nitrogen (N_2_) for 30 min to prevent uncontrolled oxidation and the self-polymerization of DA. EDC (0.388 g) and NHS (0.117 g) were incorporated into the OHA solution (pH 5.5), which was stirred for 1 h under N_2_ protection to activate the carboxyl groups of OHA. Next, 0.474 g of DA was added and the mixture was stirred under N_2_ at 25°C for 24 h. Ultimately, the solution was dialyzed (MWCO 3500) for 3 days using deionized water to eliminate unreacted impurities, followed by freeze-drying for further use. The formation of OHA and OHA-DA was confirmed through ^1^H NMR and UV–vis spectroscopy. The degree of substitution of the aldehyde group in OHA was assessed using the hydroxylamine hydrochloride titration method. The quantitative measurement of DA content in OHA-DA was conducted using a UV–vis spectrometer by assessing the absorbance at 280 nm in comparison with a DA standard.

### Preparation of OHA-DA/PAM/BC multifunctional hydrogel

The preparation process of the conventional pure PAM hydrogel was conducted as follows: 315 mg of AM monomers were dissolved in 600 μl of deionized water. After 30 min of continuous stirring, 150 μl of APS solution (50 mg/ml) and 150 μl of BIS solution (2 mg/ml) were added sequentially. The mixture was then degassed with N_2_, and 3 μl of TEMED was quickly introduced, followed by a reaction at 60°C for 4 h, resulting in the formation of the PAM hydrogel. For the OHA-DA/PAM/BC hydrogel, 10% OHA-DA solutions of varying volumes and BC suspensions at different mass concentrations (0.3%, 0.5%, 0.8%, 1% and 2%) were incorporated into the PAM hydrogel system in place of aqueous solutions (as shown in Table 1), thereby yielding a BC nano-reinforced multifunctional hydrogel (OHA-DA/PAM/BC).

**Table 1.** The compositions of various hydrogels

Hydrogel code	AM (mg)	OHA-DA (10%, μl)	DI (μl)	BC (mg)	APS (μl)	BIS (μl)	TEMED (μl)
PAM	315	0	600	–	150	150	3
OHA-DA0.3/PAM		27	573	–			
OHA-DA0.5/PAM		45	555	–			
OHA-DA0.7/PAM		63	537	–			
OHA-DA1.0/PAM		90	510	–			
OHA-DA1.3/PAM		117	483	–			
OHA-DA0.3/PAM/BC3		27	573	1.719			
OHA-DA0.3/PAM/BC5		27		2.865			
OHA-DA0.3/PAM/BC8		27		4.584			
OHA-DA0.3/PAM/BC10		27		5.730			
OHA-DA0.3/PAM/BC20		27		11.460			

### Characterization

After being frozen in liquid nitrogen and subsequently dried in a freeze dryer, the microstructures of the hydrogel samples were examined using a scanning electron microscope (SEM, JSM-7800F, JEOL, Japan). Prior to observation, each hydrogel sample was bisected, and the resulting cross-sections were treated as planes. The samples were then coated with a thin layer of platinum. To confirm the presence of specific chemical groups, the samples were analysed using Fourier transform infrared (FTIR, Nicolet iS50) spectroscopy over a wavelength range of 400–4000 cm^−1^, as well as Raman spectroscopy (HORIBA Jobin Yvon S.A.S., France).

### Mechanical properties of the hydrogels

Tensile and compression tests were conducted on the hydrogels at ambient temperature using a universal testing apparatus (CMT1202, Zhuhai SUST, China) equipped with a 100 N load cell. In the compression experiment, the hydrogels were fabricated into cylindrical shapes, measuring 11 mm in diameter and 9 mm in height, with a compression speed of 5 mm/min applied. The failure strain and stress were recorded at the point of initial crack formation point. The compressive stress (*σ*) was calculated using the formula *σ* = *F*/*S*, where *F* represents the compressive force (N) and *S* indicates the original cross-sectional area (m^2^). The compressive strain (*ε*) was calculated using the formula as (*H* − *H*_0_)/*H*_0_ × 100%, where *H*_0_ signifies the initial height of the hydrogel (mm) and *H* represents the final height after failure (mm). The compressive elastic modulus was assessed by determining the slope of the linear region of the stress–strain curve within the 0–10% strain range. In the tensile testing, the hydrogels were sectioned into rectangular strips measuring 25 mm in length, 10 mm in width and 3 mm in thickness, subjected to a stretching speed of 100 mm/min. The tensile stress (*σ*) was derived using the equation *σ* = *F*/*S*, with *F* denoting the tensile force (N) and *S* as the original cross-sectional area (m^2^). The tensile strain (*ε*) was defined as (*L* − *L*_0_)/*L*_0_ × 100%, where *L*_0_ corresponds to the hydrogel's initial length (mm) and *L* represents the length after breaking (mm). For each hydrogel composition, the compression and tensile measurements were repeated at least five times to present the results as the mean ± standard deviation.

### Rheological tests

The rheological characteristics of the hydrogel samples, including PAM, OHA-DA0.3/PAM and OHA-DA0.3/PAM/BC8, were evaluated at temperatures of 25°C and 37°C using a rheometer (Discovery Hybrid Rheometer-1, Waters Corporation, USA). The samples were positioned between two parallel plates (diameter: 40 mm, gap: 500 μm), and both the storage modulus (*G*′) and loss modulus (*G*″) were evaluated at a strain of 1% and within a frequency range of 1–100 rad/s. To further investigate the viscoelastic characteristics of the hydrogels, a time sweep experiment was conducted over a duration of 600 s, with the strain and frequency parameters maintained at 1% and 1 Hz, respectively.

### Injectability and self-healing performance test

To assess injectability, the OHA-DA0.3/PAM/BC8 hydrogel was loaded into a pipette and subsequently extruded. Photographs were taken to document the injection process and the appearance of the resulting hydrogel. Furthermore, both the macroscopic and microscopic self-healing properties of the hydrogels were examined to evaluate their self-healing capabilities. For the qualitative observation test, two disk-shaped OHA-DA0.3/PAM/BC8 hydrogels (diameter of 10 mm and thickness of 3 mm) were prepared, with one dyed using rhodamine B for observation purposes. Following this, the two hydrogels were halved. The semicircular hydrogels, displaying different colors, were then united and allowed to heal naturally at room temperature without any external stimuli. Both macroscopic and microscopic images were captured to document the self-healing process. In the quantitative analysis of self-healing, the tensile properties of the self-healed OHA-DA0.3/PAM, OHA-DA0.3/PAM/BC8 hydrogels and the original hydrogels were measured. The self-healing efficiency was calculated using the formula SH = *σ*/*σ*_0_ × 100%, where *σ*_0_ denotes the initial tensile strength and *σ* represents the tensile strength after self-healing.

### Adhesion properties of the hydrogels

The adhesive performance of OHA-DA0.3/PAM and OHA-DA0.3/PAM/BC8 hydrogels on different substrates was evaluated through lap-shear tests executed with a universal testing machine. Briefly, the hydrogels (10 × 10 × 3 mm) were adhered separately to different substrates (glass, paper, rubber, plastic, iron, and pig skin), with an adhesive area of 10 × 10 mm. Prior to testing, the hydrogel samples were kept in close contact with the substrates for 30 min to ensure optimal adhesion. The tensile lap-shear tests were conducted at a crosshead speed of 50 mm/min under room temperature conditions. The adhesive strength (kPa) was determined by dividing the peak load (N) by the adhesive area (m^2^). Each test group was evaluated five times. The hydrogel affixed to the hand underwent continuous hydrodynamic flushing to assess its adhesion stability under dynamic conditions, encompassing evaluations on wet surfaces and in underwater environments.

### Swelling test of hydrogels

Freeze-dried PAM, OHA-DA0.3/PAM and OHA-DA0.3/PAM/BC8 hydrogels (with a diameter of 10 mm and a thickness of 3 mm) were incubated in deionized water at 37°C. At predetermined time intervals (days 0, 1, 2, 3 and 4), the hydrogels were taken out from the solutions, and excess surface water was blotted away using filter paper. Subsequently, the wet weights of hydrogels were measured. The experimentation continued until the weights of all hydrogels stabilized, after which the materials were freeze-dried and weighed. The swelling rate (SR) was determined by the equation SR = *W*_s_/*W*_0_, while the average water content was obtained using the formula (*W*_s_ − *W*_d_)/*W*_s_, in which *W*_s_ indicated the weight of swollen hydrogels at each time interval, *W*_d_ represented the weight of the dried swollen hydrogel, and *W*_0_ indicated the initial weight of the dried hydrogel at day 0. This test was conducted in triplicate.

### Antibacterial test *in vitro*

The antibacterial characteristics of PAM, OHA-DA0.3/PAM, OHA-DA0.3/PAM/BC8 hydrogels were examined against *Escherichia coli* (*E. coli*, a common gram-negative bacteria) and *Staphylococcus aureus* (*S. aureus*, a typical gram-positive bacteria) in this study, commercial 3M wound dressing was used as positive control. Luria-Bertani agar and Luria-Bertani broth served as the culture media. Initially, all hydrogel samples were sterilized through UV irradiation for 30 min before the antibacterial assay. Subsequently, the microorganisms were subcultured in the appropriate culture media to verify their purity. A suspension of *E. coli* and *S. aureus* was then spread across the culture medium for inoculation. The hydrogels were positioned in various locations on the plate, which was then incubated at 37°C. Inhibition zones were observed at 12 and 24 h. To further investigate the antibacterial ability of the hydrogels, precultured *E. coli* and *S. aureus* were cultured in a fluid medium containing the experimental hydrogels while utilizing an automated shaker set to 37°C. The optical densities (OD600) of the bacterial cultures were assessed at various incubation times.

### Hemolysis assay

The *in vitro* hemolytic potential of hydrogels was systematically evaluated in accordance with ASTM-compliant protocols to assess blood compatibility. Venous blood was collected in EDTA-anticoagulated tubes and subjected to three sequential centrifugation cycles (3000 rpm for 10 min), followed by isotonic PBS washing to isolate purified erythrocyte concentrates. After anticoagulation, the erythrocyte suspension was standardized to 2% (w/v) in PBS at pH 7.4 for experimental use, with the parallel preparation of positive (deionized H_2_O) and negative (PBS) controls. Individual hydrogel specimens (*n* = 3 per formulation) were incubated with 1 ml aliquots of the erythrocyte suspension at 37°C for 2 h under static conditions. Post-incubation supernatants were obtained through centrifugation (3000 rpm for 10 min) and analysed spectrophotometrically at 540 nm using a multimode microplate reader. Macroscopic hemolytic responses were documented through high-resolution imaging of the centrifuged pellets. Hemolysis indices were calculated via normalized absorbance ratios using the following equation: hemolysis ratio (%) = (*h*1—*h*0)/(*h*100—*h*0) × 100%, where *h*0, *h*1 and *h*100 represent the absorbance values of the negative control (PBS), test sample and positive control (H_2_O), respectively. All experimental procedures were conducted in triplicate to ensure statistical validity.

### *In vivo* wound healing and histological analysis

*In vivo* experiments on wound healing were conducted in mice using full-thickness skin defect models, following established protocols. All procedures involving experimental animals received approval from the Experimental Animal Management Committee of the 960 Hospital of the People's Liberation Army (2023-095). SPF C57BL/6N mice (5–6 weeks old, weighing 25.0–30.0 g) obtained from Beijing Weitong Lihua Experimental Animal Technology Co., Ltd, were assigned randomly into four groups: Group I (PBS), Group II (PAM), Group III (OHA-DA0.3/PAM) and Group IV (OHA-DA0.3/PAM/BC8). Following anesthesia with chloral hydrate (0.4 mg/kg body weight), the fur on the dorsum of the mice was shaved, and a full-thickness skin defect measuring 8 mm in diameter was produced using tissue scissors to establish the wound defect model. The corresponding hydrogels were evenly applied to and adhered to the wound surface. Over time, the hydrogel detached naturally after drying, and the healing process was observed and photographed on days 0, 4, 7, 11 and 14 using a digital camera. The collected tissue samples were fixed in paraformaldehyde and dehydrated using a gradient dehydrator (WHJJ, JT-12J, China). Subsequently, the samples were embedded in paraffin, sectioned into 4 μm slices and stained with hematoxylin & eosin (H&E) and Masson’s trichrome stain.

### Statistical analysis

The mean and standard deviation were used to express the statistical data. To ensure accuracy, each experiment was conducted at least three times. For the statistical analysis, one-way ANOVA was performed using the Origin 2021 statistical software. Differences between the groups were regarded as statistically significant at *P < 0.05, **P < 0.01 and ***P < 0.001 were categorized as highly significant.

## Results

### Synthesis and characterization of OHA-DA/PAM/BC hydrogel

In this study, polysaccharide HA was chosen not only for its biocompatibility but also for its ease of modification with functional groups. Initially, aldehyde groups were successfully introduced into the HA backbone via NaIO_4_ oxidation, resulting in OHA. To enhance the adhesion of HA-based hydrogels, inspired by mussels, the amino group in DA was reacted with the carboxyl group in OHA through a classical EDC/NHS-catalyzed reaction to produce DA-grafted OHA (OHA-DA). The specific reaction mechanisms for the syntheses of OHA and OHA-DA are illustrated in Supplementary Figure S1A. ^1^H NMR spectroscopy was conducted to confirm the synthesis of OHA and OHA-DA. As illustrated in Supplementary Figure S1B, OHA displayed a new peak at 5.0 ppm, which is indicative of the presence of an aldehyde group, in contrast to the HA sample. This observation confirms the successful oxidation of HA. The hydroxylamine hydrochloride titration method was utilized to quantify the degree of substitution of the aldehyde group, yielding an oxidation degree of 49.4%. In the ^1^H NMR spectra of OHA-DA, new proton signals appeared at 6.7 ppm from the catechol ring, and a proton peak at 2.76 ppm was attributed to a methylene (–CH_2_–) group adjacent to the catechol ring within DA, indicating successful modification of the DA graft. Furthermore, the conjugation of DA to OHA was corroborated through UV–vis absorption spectroscopy (Supplementary Figure S1C). The characteristic peak of DA was observed at 280 nm, while the OHA-DA solutions exhibited stronger absorption around 285 nm compared to the DA solution, indicating that OHA-DA retained a greater number of catechol groups, thereby establishing a solid foundation for imparting certain adhesion properties to the hydrogels. However, OHA did not exhibit a characteristic peak at 280 nm, indicating successful grafting of DA onto the OHA. Additionally, the absence of a peak at 395 nm for OHA-DA suggests that quinones were not generated due to the oxidation of catechol groups in OHA-DA during the synthesis process. According to the standard curve of DA, as depicted in Supplementary Figure S1D, there is a strong linearity in the range of 10–100 μg/ml, with a calibration equation of *y* = 0.00522*x* + 0.03049 and an *R*^2^ value of 0.99895. The catechol group content in OHA-DA was calculated, revealing a degree of DA substitution of 15.77%.

**Figure 2.**
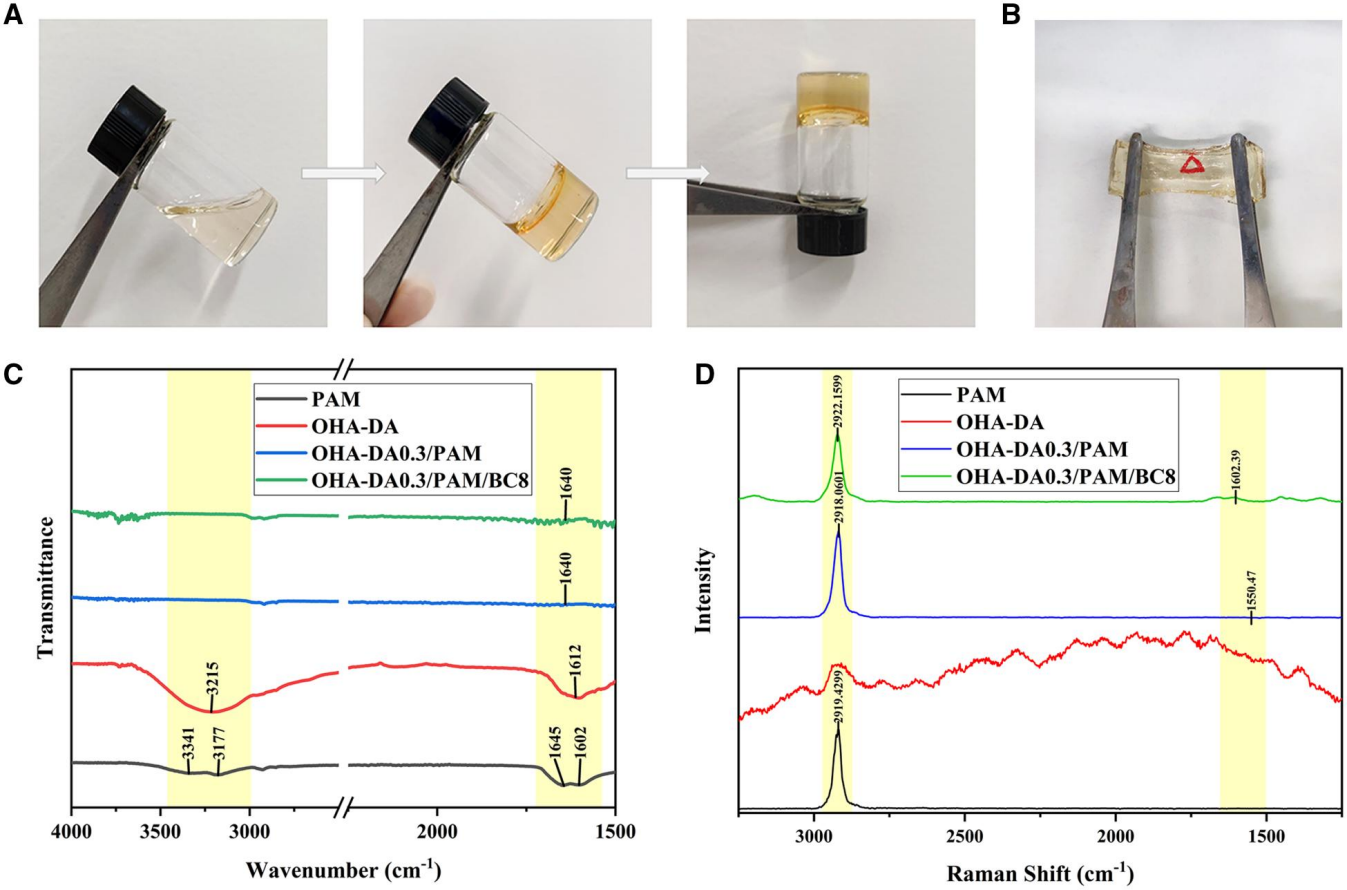
Optical images of gelation process (**A**). Transparency of the OHA-DA0.3/PAM/BC8 hydrogel (**B**). The FTIR (**C**) and Raman spectrum (**D**) of OHA-DA conjugate, PAM, OHA-DA0.3/PAM and OHA-DA0.3/PAM/BC8 hydrogels.

Subsequently, OHA-DA/PAM/BC multifunctional hydrogels were synthesized using a straightforward one-pot method, following a 4-h mixing of the reactants at 60°C (Figure 2A). As illustrated in Figure 2B, a triangle beneath the hydrogel was visible, signifying good transparency. Conventional DA can readily oxidize spontaneously, resulting in the formation of black PDA [43–45]. In contrast, chemical grafting is used to graft the DA monomer onto the OHA chain to form the three-dimensional network structure, which further prevents its oxidation and polymerization. This feature in turn ensures the transparency of the hydrogel, which is convenient for observing wound healing. To further validate the gel-forming mechanism of the hydrogels, the FTIR and Raman spectra were obtained. Figure 2C shows the FTIR spectra of the PAM, OHA-DA, OHA-DA0.3/PAM and OHA-DA0.3/PAM/BC8 hydrogels. In the pure PAM hydrogel, a characteristic absorption peak of free –NH_2_ was found at 3341 cm^−1^, alongside an associated –NH_2_ peak at 3177 cm^−1^, a C=O carbonyl group peak at 1645 cm^−1^ and an N–H bending vibration at 1602 cm^−1^. In OHA-DA, the C=O stretching vibration of the aldehyde group was observed at roughly 1612 cm^−1^. However, in OHA-DA0.3/PAM and OHA-DA0.3/PAM/BC8 hydrogels, the amino peak of PAM and the aldehyde peak of OHA-DA disappeared, but a new characteristic peak corresponding to the imine bond appeared in both hydrogels at 1648 cm^−1^, indicating that the Schiff base-crosslinked OHA-DA0.3/PAM and OHA-DA0.3/PAM/BC8 hydrogels were successfully prepared. The Raman spectrum of PAM further shows that the C=N stretching vibration peak of the Schiff base bond appears at approximately 1600 cm^−1^ in the OHA-DA0.3/PAM and OHA-DA0.3/PAM/BC8 hydrogels, which further proves the formation of Schiff base bonds (Figure 2D). The results showed the hydrogels were synthesized through a combination of radical polymerization of AM monomers, dynamic Schiff reaction between OHA-DA and PAM, and dynamic hydrogen bonding among BC, OHA-DA and PAM.

### Micromorphological analysis of the hydrogels

**Figure 3.**
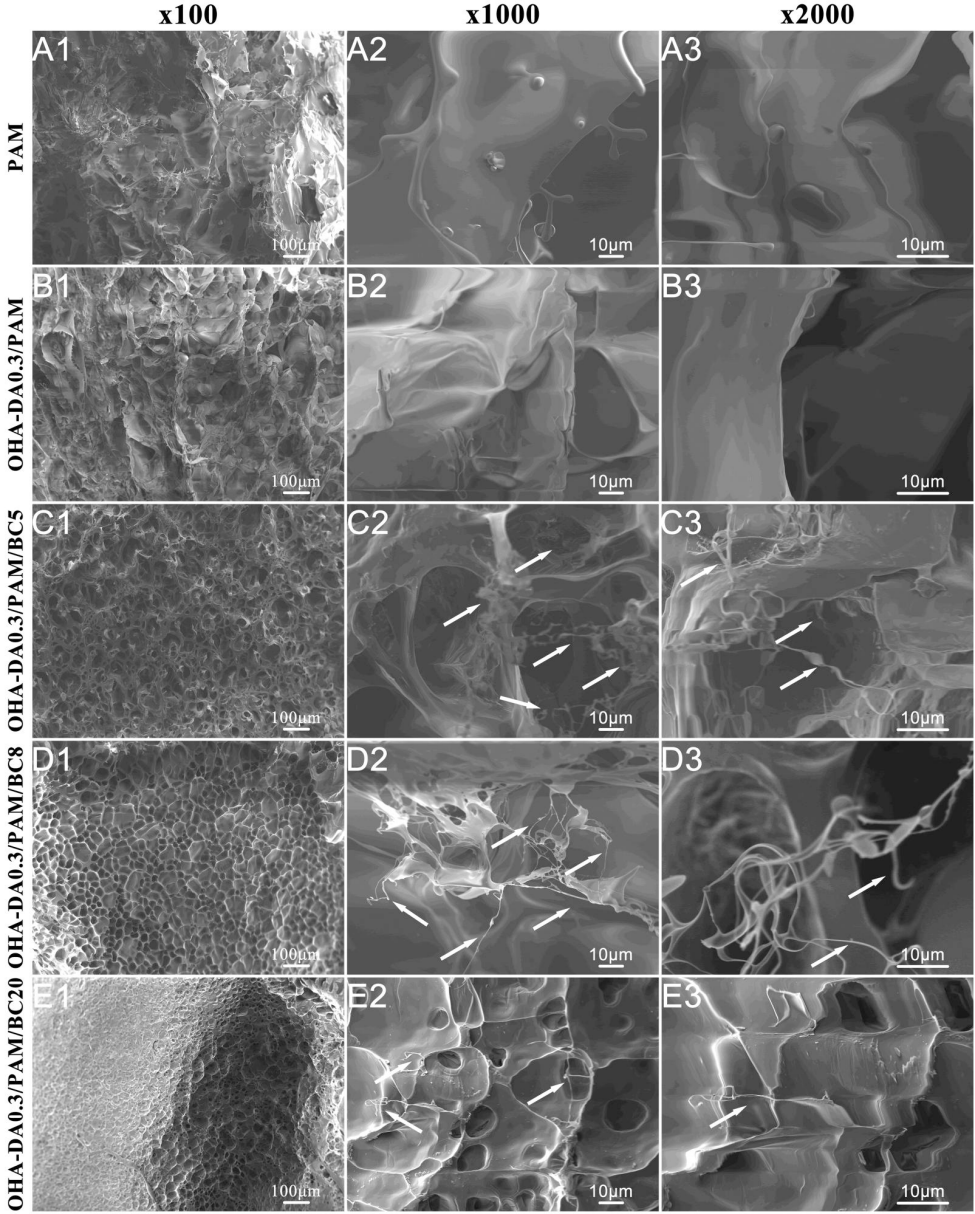
SEM images of PAM, OHA-DA0.3/PAM, OHA-DA0.3/PAM/BC5, OHA-DA0.3/PAM/BC8 and OHA-DA0.3/PAM/BC20 hydrogels, (**A1–E1**, ×100), (**A2–E2**, ×1000), (**A3–E3**, ×2000), the arrows show interwoven BC microfibrils, which simulate the microstructure of lotus-fiber.

To investigate the effect of OHA-DA and BC incorporation on the micromorphology of composite hydrogels, we examined PAM, OHA-DA0.3/PAM, OHA-DA0.3/PAM/BC5, OHA-DA0.3/PAM/BC8 and OHA-DA0.3/PAM/BC20 using SEM (Figure 3). Each lyophilized hydrogel exhibited a porous, interconnected three-dimensional mesh framework that enhanced the uptake of wound exudates. In contrast to the irregularly collapsed pore microstructure observed in the pure PAM hydrogel (Figure 3A), the OHA-DA/PAM and OHA-DA/PAM/BC hydrogels displayed smoother pore wall patches. Furthermore, the presence of interwoven microfibrils was distinctly visible in the OHA-DA/PAM/BC hydrogels (indicated by arrows in Figure 3C–E), which were absent in both the pure PAM and OHA-DA/PAM hydrogels. This observation further suggests that BC was successfully incorporated into the OHA-DA/PAM hydrogels. The interwoven microfibrils mimic the microstructure of lotus-fiber, which is advantageous for energy dissipation under mechanical impact. Additionally, the porous nature of these hydrogels plays a crucial role in hydrogel-based wound dressings, facilitating the transport of water vapor and allowing for the unobstructed movement of culture media and various nutrients throughout the porous framework. Consequently, a hydrogel resembling bionic lotus-fiber with a functional microstructure was successfully prepared.

### Mechanical properties of hydrogels

**Figure 4.**
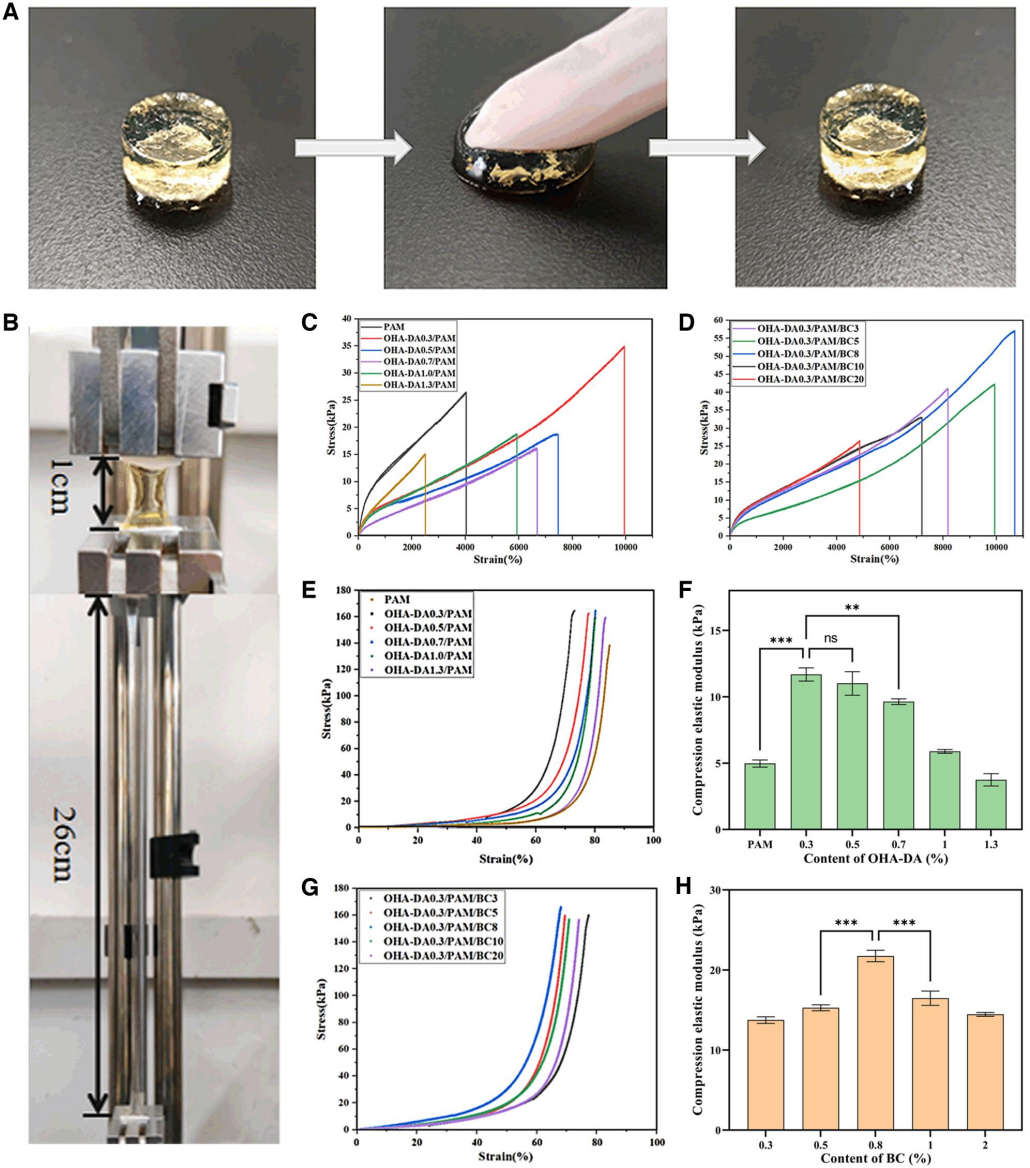
Optical photographs of OHA-DA/PAM/BC hydrogels under compression (**A**) and stretching (**B**) conditions. Tensile, compression stress–strain curves and elastic moduli of hydrogels with varying OHA-DA (**C, E, F**) and BC contents (**D, G, H**).

Hydrogels with inadequate mechanical properties are prone to tearing and peeling, which diminishes the lifespan of dressings and increases the likelihood of wound infection. Therefore, robust mechanical properties are essential for the practical application of hydrogels. Figure 4A demonstrates that the hydrogel can swiftly return to its initial state shortly after the removal of external force, as evidenced by the application of pressure with a finger. This observation indicates that the OHA-DA/PAM/BC hydrogel exhibits commendable mechanical resilience. Additionally, Figure 4B illustrates that the hydrogel can be elongated to a certain extent without fracturing, signifying its excellent toughness. A series of OHA-DA/PAM hydrogels with varying volumes of OHA-DA, as well as OHA-DA/PAM/BC hydrogels containing different amounts of BC, were prepared to quantitatively assess the effects of OHA-DA and BC concentrations on the mechanical properties of the hydrogels (Table 1).

Figure 4C presents the tensile stress–strain curves for various volumes of OHA-DA. The OHA-DA0.3/PAM hydrogel exhibited a maximum elongation at break of 9949% and a peak tensile strength of 34.73 kPa at an OHA-DA content of 0.3%, significantly surpassing the values of the pure PAM hydrogel, which recorded 4022% elongation and 26.42 kPa tensile strength. This enhancement can be attributed to the increased entanglement between the OHA-DA and PAM chains, facilitated by the presence of numerous reversible Schiff covalent bonds and hydrogen bonds. As the OHA-DA content increased to 0.5%, 0.7% and 1.0%, the fracture strain values remained higher than those of the pure PAM hydrogels but were lower than those of the OHA-DA0.3/PAM. Conversely, the tensile strength values decreased compared to the pure PAM hydrogels. This trend can be attributed to the inhibitory effect of DA molecules on the activity of the initiator APS. This inhibition subsequently impedes the free radical polymerization of AM monomers, leading to a compromised PAM network [46]. When the OHA-DA concentration was further raised to 1.3%, the significant delay in polymerization markedly diminished the synergistic interaction between the PAM networks and the PAM/OHA-DA networks, leading to a notable reduction in both tensile strength and fracture strain of the OHA-DA1.3/PAM hydrogel, which fell below the values of the pure PAM hydrogel. These experimental results align with those of a previous study [47, 48]. Therefore, the OHA-DA content was fixed at 0.3% to prepare the BC-incorporated OHA-DA/PAM hydrogels. As a type of nano-cellulose, BC exhibits high strength and rigidity, which can significantly enhance the mechanical properties of hydrogels. The PAM/BC hydrogels without OHA-DA were also prepared to clarify the individual contribution of BC. After the introduction of BC, the tensile strength of the PAM/BC8 hydrogel developed in this study was higher (66.54 kPa), although the elongation at break was lower, measuring only 708% (as shown in Supplementary Figure S2A). This phenomenon can be attributed to the fact that the incorporation of BC enhances the rigidity of the material, thereby reducing its ductility and making it more susceptible to fracture under tensile stress. While BC improves the material's tensile strength, it simultaneously restricts its ductility. As illustrated in Figure 4D, the tensile stress–strain curves of the OHA-DA0.3/PAM hydrogels with varying BC contents were synthesized. It is evident that the tensile properties of the OHA-DA/PAM/BC hydrogels, which featured a lotus-fiber simulation structure, were significantly enhanced upon the addition of a specific amount of BC, particularly at a BC concentration of 0.8%. The tensile strength and elongation at break achieved maximum values of 57.04 kPa and 10679%, respectively. These values were substantially higher than those of the previously mentioned PAM, OHA-DA/PAM and PAM/BC hydrogel, a result attributed to the homogeneous dispersion of hydrophilic BC and the interfacial hydrogen bond interactions among BC, OHA-DA and PAM. This interaction enabled the hydrogel to absorb greater energy during stretching, thereby increasing its elongation at break. However, when the BC concentration exceeded 1.0%, both the elongation at break and tensile strength of the hydrogels decreased. This phenomenon can be explained by the increased BC content within the hydrogel, which leads to self-accumulation and aggregation, resulting in a non-uniform distribution of the three-dimensional network structure and a subsequent reduction in the mechanical properties of the hydrogel. These results indicate that the incorporation of an appropriate amount of BC into the OHA-DA/PAM hydrogel system significantly enhances the tensile properties of the hydrogels. The combination of OHA-DA and BC has been demonstrated to improve the mechanical properties of PAM hydrogel through distinct mechanisms. Specifically, OHA-DA increases the elongation at break by promoting enhanced flexibility and toughness within the network, while BC contributes to tensile strength by augmenting the strength and rigidity of the material. The synergistic interaction between these two components effectively mitigates the restrictions imposed by BC on the movement of PAM molecular chains, resulting in high tensile strength and exceptional elongation at break in the OHA-DA/PAM/BC hydrogel.

Compression plays a crucial role in the application of hydrogels for wound dressings. The impact of varying concentrations of OHA-DA and BC on the compression stress–strain curves (Figure 4E and G) and compressive elastic modulus (Figure 4F and H) of the OHA-DA/PAM/BC hydrogels was found to be similar to that of the tensile strength. At an OHA-DA content of 0.3%, the hydrogel exhibited a peak stress of 164.64 kPa and an elastic modulus of 11.68 kPa, significantly exceeding the values of the pure PAM hydrogel, which demonstrated a maximum stress of 137.20 kPa and an elastic modulus of 4.97 kPa. The compressive strength and modulus of the OHA-DA0.3/PAM/BC hydrogel initially increased before declining as the BC content rose. At a BC concentration of 0.8%, the OHA-DA0.3/PAM/BC8 hydrogel achieved a peak stress of 166.65 kPa and an elastic modulus of 21.75 kPa. These values surpassed those of the pure PAM and OHA-DA0.3/PAM hydrogels, but were lower than those exhibited by the PAM/BC8 hydrogels, which demonstrated a peak stress of 431.56 kPa and an elastic modulus of 76.47 kPa (as illustrated in Supplementary Figure S2B). This discrepancy is likely due to the enhanced flexibility of OHA-DA, which mitigated the strengthening effect of BC. In conclusion, the performance is optimized at a BC concentration of 0.8%, attributed to a harmonious balance between the strengthening effect of BC and the flexibility effect of OHA-DA at this concentration. Therefore, the hydrogels demonstrated remarkable mechanical properties when the contents of OHA-DA and BC were optimized, with OHA-DA0.3/PAM/BC8 identified as the optimal preparation condition.

### Rheological behavior of the hydrogels

The rheological properties of the hydrogels were analysed by assessing their storage modulus (*G*′) and loss modulus (*G*″) at both 25°C and physiological temperature (37°C). As illustrated in Supplementary Figure S3A and B, the *G*′ values of the PAM, OHA-DA0.3/PAM and OHA-DA0.3/PAM/BC8 hydrogels consistently exceeded the G″ values and displayed stability throughout the frequency range, indicating superior mechanical performance. Furthermore, the G′ value of the OHA-DA0.3/PAM hydrogel was significantly greater than that of the pure PAM hydrogel at both 25 and 37°C, likely due to the incorporation of OHA-DA, which introduced Schiff and hydrogen bonds that reinforced the strength of hydrogel. The *G*′ value for the OHA-DA0.3/PAM/BC8 hydrogel was slightly higher than that of the OHA-DA0.3/PAM hydrogel at both 25 and 37°C, implying that the addition of BC further enhanced the strength. The results from the rheological evaluations align with the mechanical test results previously mentioned. Additionally, both the *G*′ and *G*″ values remained relatively unchanged with the extension of angular frequency, indicating good stability. As shown in Supplementary Figure S3C and D, when the strain and frequency were maintained at 1% and 1 Hz, respectively, the *G*′ values of PAM, OHA-DA0.3/PAM and OHA-DA0.3/PAM/BC8 were significantly higher than *G*″ at 600 s. Furthermore, no notable effects on gelation were observed at 25 and 37°C, suggesting their promising potential applications in biomedical fields, particularly for wounds healing.

### Injectability and self-healing properties of hydrogels

**Figure 5.**
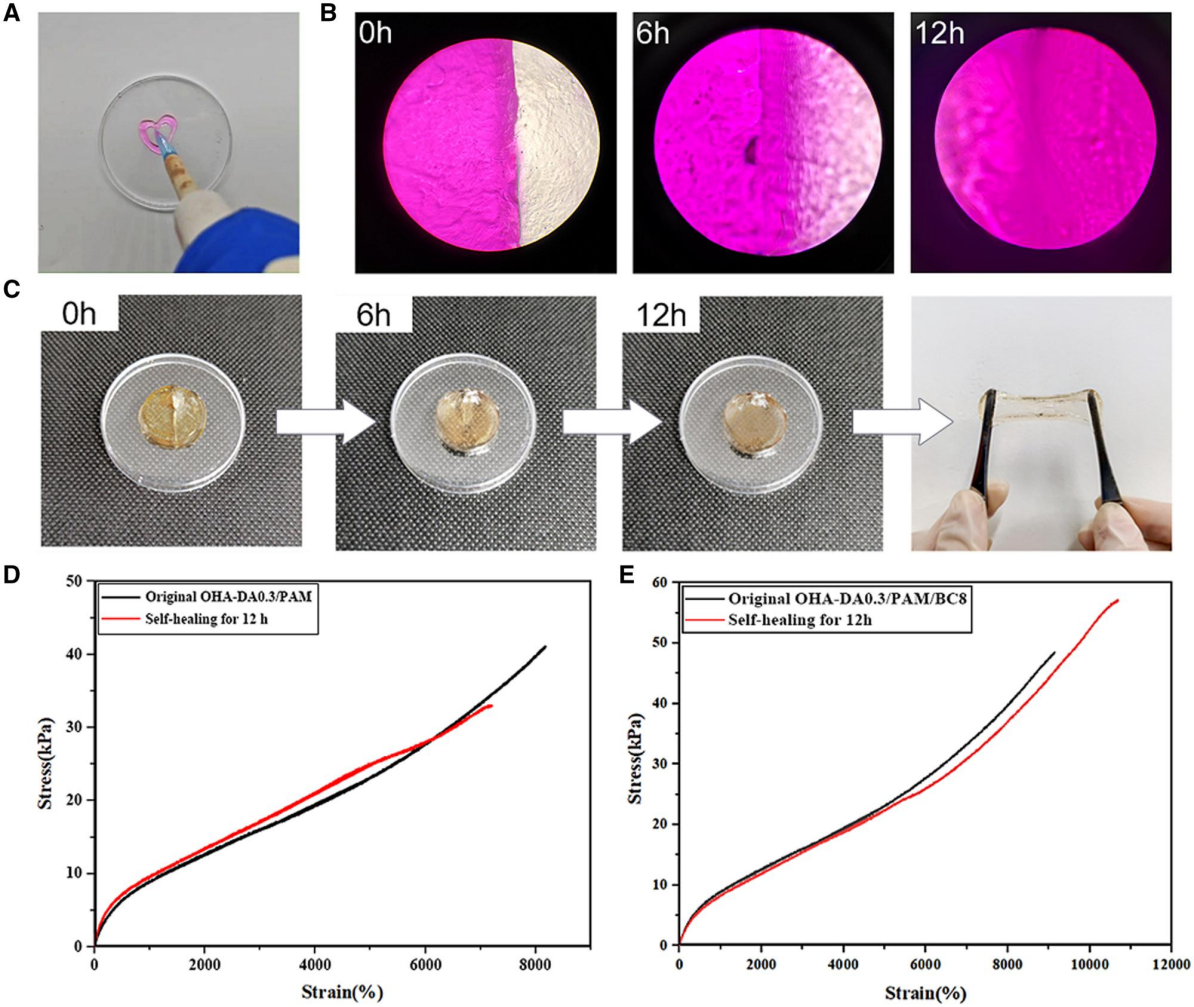
Injectable performance (**A**), microscopic (**B**) and macroscopic (**C**) self-healing behavior of OHA-DA0.3/PAM/BC8 hydrogel. Tensile stress–strain curves for both the original and the self-healed OHA-DA0.3/PAM (**D**) and OHA-DA0.3/PAM/BC8 (**E**) hydrogels.

Figure 5A shows that the OHA-DA0.3/PAM/BC8 hydrogel could be continuously extruded through a 1 ml pipette with ease. Furthermore, the hydrogel fragments rapidly return to their gel state and merge into a cohesive structure, demonstrating excellent injectability. This remarkable injectability is attributed to the shear-thinning characteristics of the dynamic Schiff base linkages and the hydrogen crosslink network inherent in the hydrogels. When pressure is applied, the hydrogel is easily dissociated as it is expelled from the pipette, allowing the disintegrated hydrogel to flow like a liquid through the slender pipette. Upon the removal of pressure, the linkages rapidly re-associate, transforming the severed hydrogel into a unified structure outside the pipette, suggesting potential for promising biomedical applications. Traditional PAM hydrogels do not exhibit self-healing properties and are susceptible to be damaged from external forces. Hence, the injectable self-healing hydrogel can be injected into a variety of shapes and can repair itself if it breaks, which extends the lifespan of the dressing and reduces the risk of infection.

In addition to their favorable mechanical behavior and injectability, the designed OHA-DA/PAM/BC hydrogels also exhibited self-healing capabilities due to the dynamic Schiff base linkages and hydrogen bonds present in their network structure. As shown in Figure 5B and C, the two distinct semicircular hydrogels were combined at room temperature without any external stimuli. After a 12-h healing period, the cut marks vanished, and the hydrogel could be stretched to a certain length perpendicular to the cutting surface without fracturing, showcasing the remarkable self-healing characteristics of the hydrogel. The self-healing functions of the hydrogels developed in this experiment were derived from the dynamic Schiff covalent cross-linking involving the aldehyde group from OHA-DA and the amino group from AM, along with the reversible hydrogen bonds formed among OHA-DA, PAM and BC. To further quantify the self-healing capabilities of the OHA-DA0.3/PAM and OHA-DA0.3/PAM/BC8 hydrogels, tensile stress–strain tests were performed after self-healing and the results were contrasted with those of the original hydrogels. As depicted in Figure 5D and E, the healed hydrogels displayed a stress–strain curve similar to that of the original samples. The initial maximum tensile strength of OHA-DA0.3/PAM hydrogel was 41.05 kPa, the tensile strength after 12 h healing can reach 33.02 kPa, resulting in a self-healing rate of 80.44%. Similarly, the self-healing rate of the OHA-DA0.3/PAM/BC8 hydrogel was 84.74%, indicating that the self-healing properties were enhanced with the introduction of BC. This finding suggests that the abundant hydroxyl groups on the surface of the BC fibers improved the hydrogen bonding of the OHA-DA0.3/PAM/BC8 hydrogel, which is essential for the restoration of its mechanical properties.

### Adhesive properties of hydrogels

**Figure 6.**
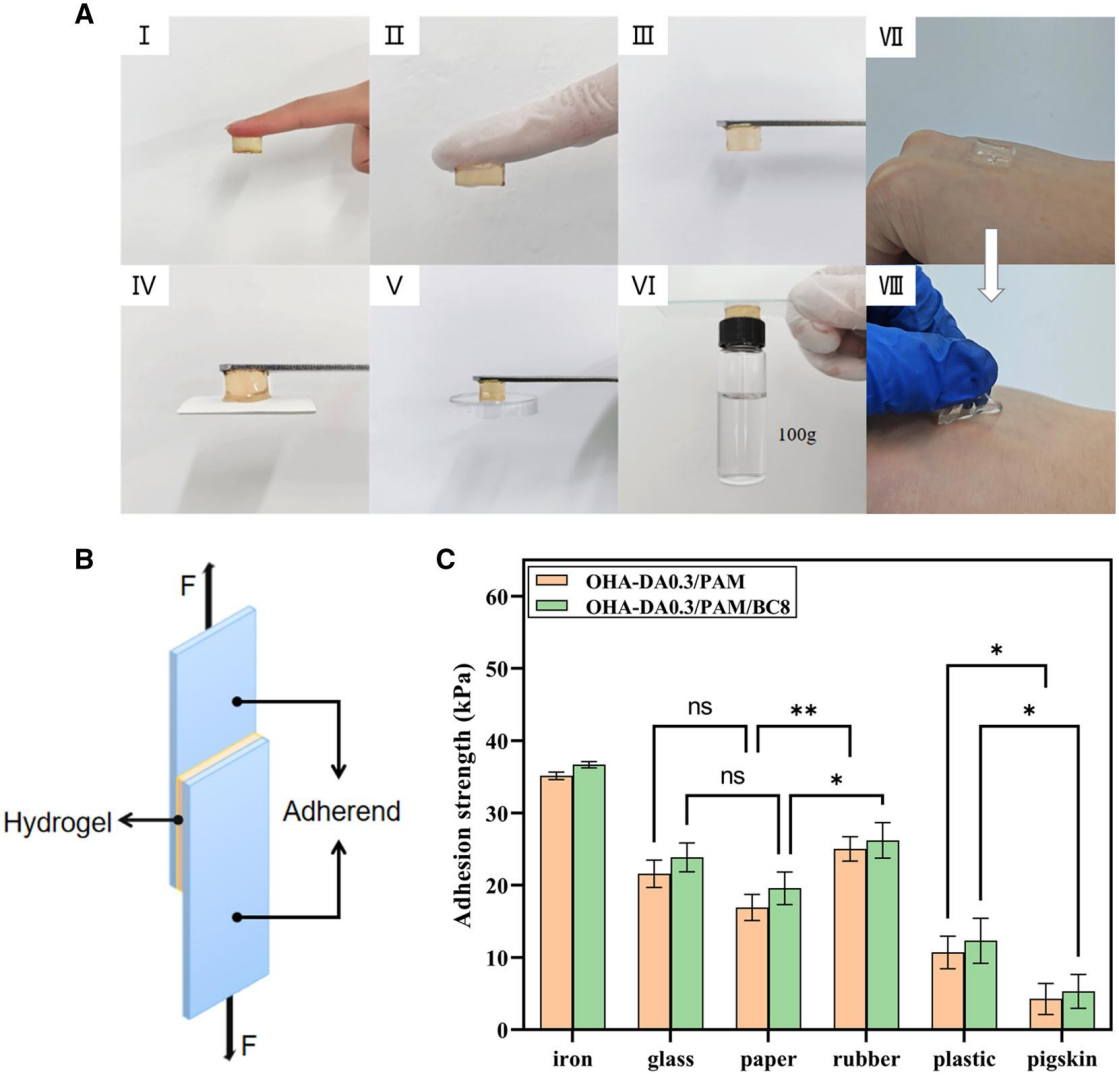
Adhesive properties of OHA-DA/PAM/BC hydrogel adhering to various materials, including fingers, rubber gloves, iron metal, paper, plastic and ceramic (**A**). Schematic of the lap-shear measurement (**B**). Lap-shear adhesion strengths of OHA-DA0.3/PAM and OHA-DA0.3/PAM/BC8 hydrogels (**C**).

As illustrated in Figure 6A (I, II, III, IV, V, VI), the OHA-DA/PAM/BC hydrogels can firmly adhere to diverse hydrophilic and hydrophobic substrates, including skin, rubber gloves, iron metal, paper, plastic and ceramics, without requiring any special surface treatment. Notably, the OHA-DA/PAM/BC hydrogel can support weights of up to 100 g without the need for additional adhesives. When employed as wound dressings, hydrogels must exhibit both adhesiveness and ease of removal to prevent further injury. Consequently, the process of removing hydrogel dressings from the skin was investigated. As depicted in Figure 6A (VII, VIII), these multifunctional hydrogels can be easily detached from the skin without leaving any residue or causing allergic reactions. As demonstrated in the video provided in the Supporting Information, the hydrogel exhibits excellent adhesion stability even under dynamic conditions, including tests conducted on wet surfaces and in underwater environments. This further substantiates its potential as a wound dressing. The adhesive mechanism of these hydrogels on various substrates is attributed to the dissociative –NH_2_, carboxyl, hydroxyl and catechol groups on their surfaces, which facilitate the formation of strong covalent and non-covalent bonds (including hydrogen bonds, coordination or van der Waals interactions) with the substrate interface. Effective adhesion to tissue allows the hydrogel to closely conform to the wound, thereby acting as a protective barrier against contamination from the external environment.

To further quantitatively assess the adhesion strength of the hydrogels to various materials, adhesion lap-shear tests were conducted on the OHA-DA0.3/PAM and OHA-DA0.3/PAM/BC8 hydrogels using a universal mechanical tester (Figure 6B). As shown in Figure 6C, the adhesion strength of the OHA-DA0.3/PAM/BC8 hydrogel was approximately 36.69 kPa for metal (iron sheet), 23.86 kPa for glass, 19.58 kPa for paper, 26.21 kPa for rubber, 12.32 kPa for plastic and 5.32 kPa for pigskin. These values were slightly higher than those of the OHA-DA0.3/PAM hydrogel, indicating that the incorporation of BC enhanced hydrogen bonding. Among the tested materials, this hydrogel demonstrated the highest adhesive strength to iron sheets, likely due to the numerous active groups present on the metal surface that interact with the abundant free catechol groups in the hydrogel. Conversely, the adhesion strength of the hydrogel to pigskin was the lowest, likely attributed to the high fat content on the surface of pigskin, which resulted in an increased presence of hydrophobic groups and consequently created a hindering effect. Furthermore, the adhesion strength of the OHA-DA/PAM/BC hydrogel in this study was much higher than those of commercially available fibrin glues (EVICEL Fibrin Sealant, approximately 3 kPa [16]), suggesting that the hydrogels have potential applications in wound repair.

### Swelling properties of hydrogels

Supplementary Figure S4 illustrates the swelling rates of various hydrogels in aqueous solutions. All hydrogels achieved swelling equilibrium within approximately 48 h. Notably, the swelling rates and average water content of the OHA-DA0.3/PAM and OHA-DA0.3/PAM/BC8 hydrogels were significantly higher than those of the PAM hydrogels. This enhancement is likely attributed to the abundance of hydroxyl and aldehyde groups present in the OHA-DA chains and BC, which render the OHA-DA/PAM and OHA-DA/PAM/BC hydrogels highly hydrophilic, thereby contributing to their superior swelling capacity. Conversely, the addition of BC resulted in a slight reduction in swelling rate and average water content, potentially due to the formation of additional hydrogen bonds and an increase in the cross-linking density of the OHA-DA0.3/PAM/BC8 hydrogel. The results of the swelling tests align with the mechanical property test findings discussed previously. Swelling in aqueous solutions is a critical characteristic of hydrogels utilized in wound dressings. During the swelling process, the hydrogel's volume expands rapidly as water molecules permeate the hydrogel network until the stretching of the molecular chains is impeded, at which point the volume expansion slows. Ultimately, the weight stabilizes, indicating that swelling equilibrium has been reached. This outcome suggests that the hydrogel can effectively absorb wound exudates and mitigate conditions conducive to the proliferation of harmful bacteria, thereby enhancing its suitability for wound dressing applications.

### *In vitro* antibacterial evaluation

**Figure 7.**
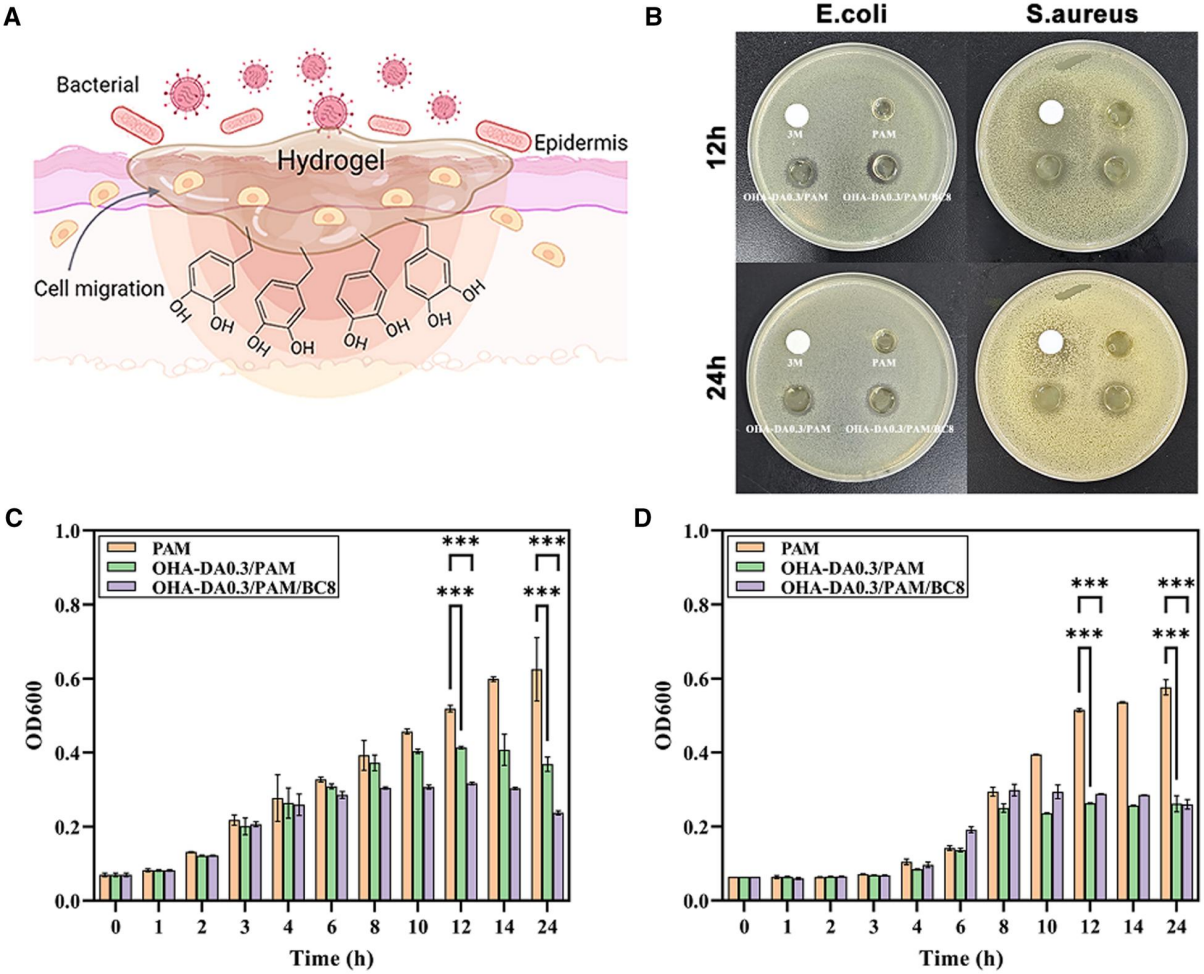
Antimicrobial scheme of hydrogel wound dressings (**A**) (created with BioRender.com). Digital photographs depicting surviving colonies of *E. coli* and *S. aureus* following exposure to 3M dressing, PAM, OHA-DA0.3/PAM and OHA-DA0.3/PAM/BC8 hydrogels for 12 and 24 h (**B**). Growth curves of *E. coli* (**C**) and *S. aureus* (**D**) over time in culture when exposed to PAM, OHA-DA0.3/PAM and OHA-DA0.3/PAM/BC8 hydrogels.

*Escherichia coli* and *Staphylococcus aureus* were employed to assess the antibacterial efficacy of the OHA-DA0.3/PAM and OHA-DA0.3/PAM/BC8 hydrogels. The antibacterial capacity of these hydrogels was demonstrated through the antibacterial zones observed on two solid media after 12 and 24 h of incubation (Figure 7B). In contrast, the pure PAM hydrogel failed to inhibit bacterial growth, as it did not form an inhibition ring. 3M wound dressings exhibit limited antibacterial efficacy against *S. aureus* during the initial application period, demonstrating poor sustained antimicrobial performance and negligible effectiveness against *E. coli*. The zones of inhibition for *E. coli* and *S. aureus* after 12 h were 13.5 mm for the OHA-DA0.3/PAM samples, while the OHA-DA0.3/PAM/BC8 formulation exhibited inhibition zones of 14 and 17 mm, respectively. This indicates that both OHA-DA0.3/PAM and OHA-DA0.3/PAM/BC8 hydrogels demonstrated significant antibacterial activity. These results suggest their capacity to effectively hinder bacterial invasion, highlighting their potential for wound dressing applications. Furthermore, the inhibition zone of the OHA-DA0.3/PAM/BC8 hydrogel was the largest among the tested samples, indicating its superior bacteriostatic properties. To further investigate the antibacterial activity of the OHA-DA0.3/PAM and OHA-DA0.3/PAM/BC8 hydrogels, the bacterial growth curves of *E. coli* and *S. aureus* co-cultured with these hydrogels were analysed. The OD600 values of the bacterial liquid media containing various hydrogels were recorded over time. As illustrated in Figure 7C and D, the OD600 values of both bacterial strains increased over time while they proliferated freely in the incubated PAM hydrogel. However, a notable decrease in the OD600 values of both *E. coli* and *S. aureus* was observed after incubation with the OHA-DA0.3/PAM and OHA-DA0.3/PAM/BC8 hydrogels, indicating their effective antibacterial properties. The antibacterial properties of these hydrogel materials can be attributed to the presence of phenolic hydroxyl (–OH) and amino (–NH_2_) functional groups within their molecular structures. These active groups possess the ability to interact specifically with the phospholipid bilayer of the bacterial cell membrane, leading to the disruption of membrane integrity through hydrogen bonding and electrostatic interactions. Consequently, this process inhibits the growth and proliferation of bacteria.

### *In vitro* hemolysis capacity

**Figure 8.**
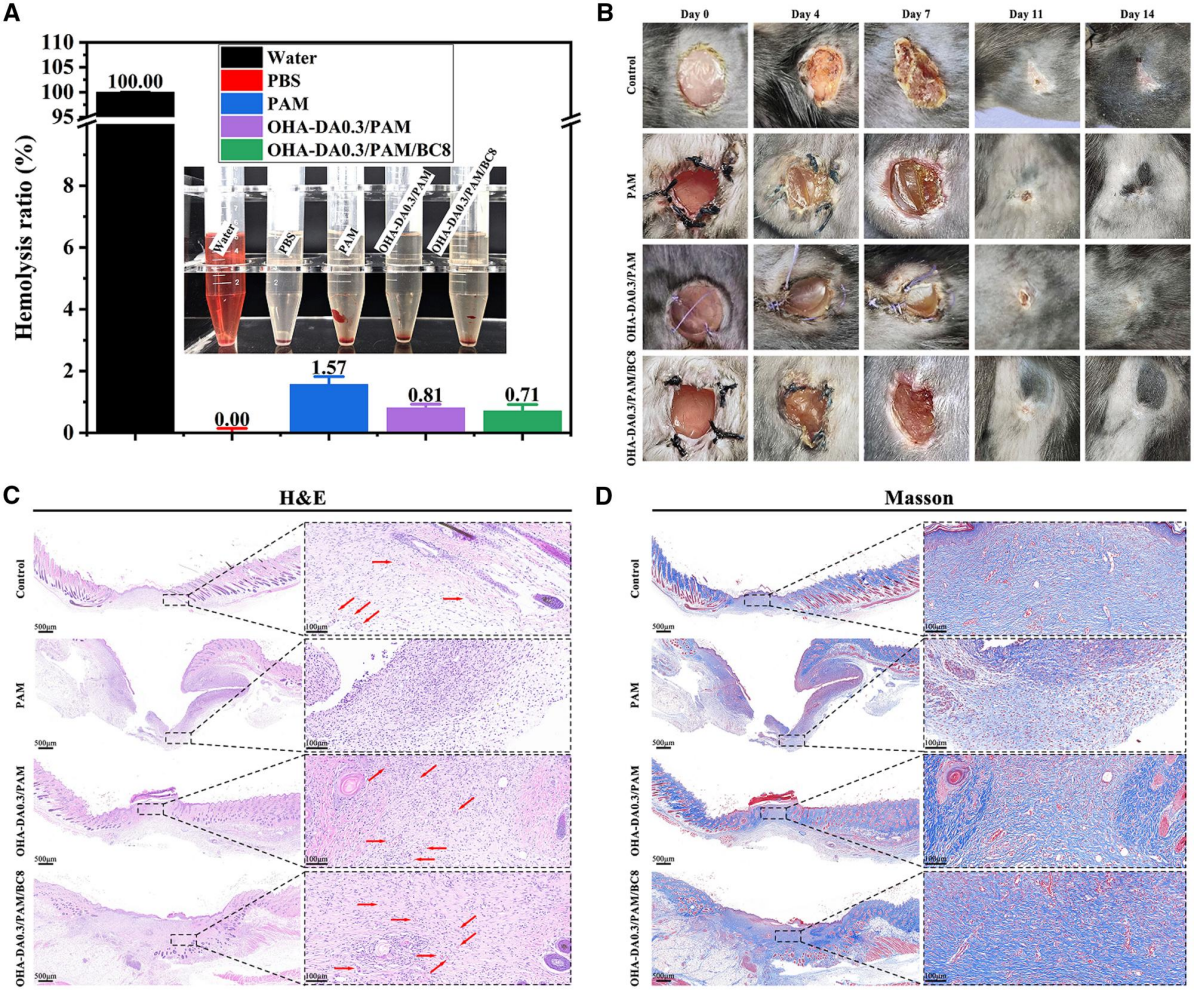
The hemolysis activity (**A**), representative photographs (**B**) of mouse wounds after treatment with control, PAM, OHA-DA0.3/PAM and OHA-DA0.3/PAM/BC8 hydrogels. H&E (**C**), and Masson's trichrome staining (**D**) of the wound tissues (the arrows indicate new blood vessels).

The hemolysis test is a method for evaluating the blood compatibility of materials. Materials are considered to meet the hemolysis standards for biomaterials if their hemolysis rate is ≤5% [49]. Since wound dressing materials are in direct contact with wound surfaces or blood, it is imperative that the prepared hydrogels comply with safety standards. In this study, we determined the hemolysis rates of PAM, OHA-DA0.3/PAM and OHA-DA0.3/PAM/BC8 hydrogels, with the results presented in Figure 8A. Following treatment with water, all red blood cells were essentially destroyed; in contrast, treatment with PBS resulted in minimal damage to the red blood cells. The treatment group with hydrogels exhibited a lower degree of damage to red blood cells. The hemolysis ratios of the hydrogels were found to be less than 5%, with PAM hydrogels showing 1.57%, OHA-DA0.3/PAM hydrogels showing 0.81% and OHA-DA0.3/PAM/BC8 hydrogels showing 0.71%. These findings indicate that the hydrogels have a low rate of red blood cell destruction and good biocompatibility, thereby meeting national safety standards.

### Ability to repair skin wounds

The animals were divided into four groups: control (PBS), PAM, OHA-DA0.3/PAM and OHA-DA0.3/PAM/BC8 hydrogel groups. Typical images of wound closure for each group at various time intervals are depicted in Figure 8B. As expected, the OHA-DA0.3/PAM and OHA-DA0.3/PAM/BC8 hydrogel groups exhibited significant healing progression over time. In contrast, the control and PAM groups displayed severe suppuration at the wound surface, with noticeable scarring persisting after 14 days of healing. Notably, the OHA-DA0.3/PAM/BC8 hydrogel group demonstrated the most favorable healing conditions among them. The addition of BC markedly enhanced wound healing, with wounds nearly disappearing after 14 days and covered by new hair.

As illustrated in Figure 8C, H&E staining indicated that the OHA-DA0.3/PAM/BC8 hydrogel considerably facilitated the development of well-organized granulation tissue at the injury site by day 14. The role of endothelial cells is vital in creating tube-like structures during angiogenesis, which is an essential process in wound healing. H&E staining demonstrated that the OHA-DA0.3/PAM/BC8 group exhibited the narrowest wound width, most granulation tissue and highest degree of angiogenesis when compared to the control group and other hydrogels, correlating with the wound contractions observed in the optical images. During the repair maturation phase, collagen deposition plays an essential role in the formation of scars, while wound contraction is facilitated by the cross-linking between fibroblasts and collagen. Masson trichrome staining was performed to visualize newly formed collagen deposits in the regenerated skin. Figure 8D displays the Masson staining image of the wound at 14 days, where the collagen in the tissue appeared blue. The intensity of the blue color in the healed tissue was markedly stronger than that of the unhealed wound. Interestingly, the accumulation of collagen in the OHA-DA0.3/PAM/BC8 hydrogel group was markedly higher than that in the other groups, especially in the control and PAM hydrogel groups. Consequently, the OHA-DA0.3/PAM/BC8 hydrogel dressing effectively enhanced wound healing and can be used as a promising option for innovative wound dressing.

## Discussion

In modern medicine, wound care is a crucial component for promoting healing and preventing infection [50]. Traditional wound dressings often fail to meet the diverse needs of various wound types regarding humidity, permeability and mechanical properties, making the development of new materials particularly important. To address the characteristics of infected wounds, a BC-reinforced multifunctional hydrogel (OHA-DA/PAM/BC) was developed (Figure 1). In summary, DA was initially grafted onto OHA to create DA-grafted OHA (OHA-DA). Next, the AM monomers were linked to the OHA-DA chains through a Schiff base reaction, utilizing the aldehyde functional groups in the OHA-DA chains and the amino groups in AM. Subsequently, both the connected and unbound AM monomers underwent chemical polymerization with the initiator APS, crosslinker BIS and catalyst TEMED to form the OHA-DA/PAM hydrogel. Furthermore, a BC suspension was employed, instead of an aqueous solution, as a nano-reinforcer in the OHA-DA/PAM hydrogel, resulting in enhanced hydrogen bond interactions. The chemical grafting of DA and the incorporation of BC into the hydrogel were essential for the formation of OHA-DA/PAM/BC hydrogels with superior properties.

Inspired by the structure of lotus fibers, an OHA-DA/PAM/BC hydrogel was developed, demonstrating high strength, toughness and extensibility. Lotus fibers are notably extensible, capable of stretching over 10 cm without fracturing [35]. At the microscale level, BC exhibits a helical configuration, which mimics the fiber structure of lotus fibers. It has been reported that BC, a rigid natural nanofiber, was incorporated into the network to enhance the mechanical properties of PAM hydrogel [51]. In this report, the tensile strength of the BC/PAM hydrogel was able to achieve a strain of 1300% without exhibiting visible fractures. However, this value remains significantly lower than that of the OHA-DA0.3/PAM/BC8 hydrogel, which reached a strain of 10679% in the present study. Consequently, integrating BC into hydrogels represents an effective strategy for improving their mechanical performance. In this study, the OHA-DA/PAM hydrogel featured a tightly crosslinked PAM network alongside a loosely crosslinked OHA-DA/PAM network system. The synergistic effects arising from these two network structures, combined with the design inspired by lotus-fiber, resulted in remarkable mechanical properties. The connection between the dressing and the dynamic wound site is a crucial factor in enhancing the healing capacity of hydrogel wound dressings. Drawing inspiration from the partial adhesion characteristics of catechol, this study improved the biological adhesion properties of the hydrogel materials by incorporating catechol into the hydrogel system. In contrast to conventional PAM hydrogel dressings [52], the OHA-DA/PAM/BC hydrogel developed in this research can adhere strongly to the wound site without the need for gauze or other external fixation techniques, thereby minimizing the risk of infection. Furthermore, unlike conventional DA, which readily oxidizes to form black PDA, this study employed chemical grafting to attach the DA monomer to the OHA chain. The resulting three-dimensional network structure of the hydrogel not only prevented oxidation and polymerization but also maintained the transparency of the hydrogel, facilitating the observation of wounds in wound dressing applications.

Bacterial infections can significantly impair the wound healing process. To enhance the recovery of infected wounds, various antimicrobial materials, such as silver nanoparticles [53], fullerenes [54] and antibiotics [55], have been incorporated into hydrogels for the treatment of wound infections. Although these substances exhibit strong antibacterial properties, improper use of inorganic antibacterial agents or antibiotics can lead to considerable systemic toxicity and the emergence of drug resistance, which is a critical issue that warrants attention. For instance, Diana *et al.* [56] prepared a temperature-sensitive hydrogel loaded with silver nanoparticles (AgNPs). However, nanoparticles may damage bacterial cell membranes or induce harmful changes to organelles. The catechol groups in DA conferred OHA-DA/PAM and OHA-DA/PAM/BC hydrogels with exceptional anti-*E. coli* and anti-*S. aureus* properties, thus promoting the wound healing process.

An ideal wound dressing should promote healing, resist infection and minimize irritation. The OHA-DA/PAM/BC hydrogel developed in this study demonstrated a superior wound healing effect compared to the existing PAM hydrogel. Yang *et al.* [57] created a hydrogel for wound healing by incorporating BC into a PDA/PAM hydrogel. The maximum tensile strain of their hydrogels was measured at 1047%, which was significantly lower than the results obtained in this study (10679%). The hydrogel required a treatment duration of 15 days to achieve wound healing, with residual scars still observable in the wound model. In particular, the OHA-DA/PAM/BC hydrogel formulated in this study exhibited minimal residual scarring after 14 days of wound healing. Additionally, the granulation tissue deposition in the OHA-DA/PAM/BC hydrogel group was denser, and collagen deposition was markedly improved, indicating substantial potential for use in wound dressings.

All hydrogels prepared in this study exhibited interpenetrating porous structures, which facilitated nutrient exchange, cell proliferation and tissue regeneration. They also possessed an appropriate degree of swelling, which aided in the absorption of wound exudate, the transport of nutrients and metabolites and the loading and release of drugs. More importantly, OHA-DA/PAM and OHA-DA/PAM/BC hydrogels demonstrated notable self-healing properties. After 12 h of self-healing, their self-healing rates were measured at 80.44% and 84.74%, respectively. This characteristic reduced the risk of dressing rupture, thereby decreasing the likelihood of bacterial infections. Additionally, the OHA-DA/PAM/BC hydrogels exhibited exceptional stretchability, adhesiveness, injectability and antibacterial properties.

## Conclusion

In summary, inspired by lotus-fiber and mussel, a BC-reinforced multifunctional hydrogel (OHA-DA/PAM/BC) was successfully developed, exhibiting super-stretchability, excellent self-healing, adhesiveness, injectability, antibacterial properties and minimal hemolytic activity. The tensile test results indicated that at a concentration of 0.3% OHA-DA and 0.8% BC, the maximum elongation at break for the OHA-DA/PAM and OHA-DA/PAM/BC hydrogels increased to 9949% and 10679%, respectively, compared to the pure PAM hydrogel which showed an elongation of 4022%. Furthermore, the tensile strength improved to 34.73 and 57.04 kPa for the two formulations, up from 26.42 kPa for the pure PAM hydrogel, demonstrating that the incorporation of OHA-DA and BC significantly enhanced the mechanical properties of the PAM hydrogel. Additionally, the introduction of catechol groups onto the DA imparted self-adhesive properties to the hydrogel, enabling it to adhere firmly to various surfaces without requiring additional adhesives. The chemical grafting of DA ensured the transparency of the hydrogel dressings, facilitating the observation of wound healing. Moreover, the inclusion of OHA-DA in the pure PAM hydrogel system enhanced the antimicrobial properties of the hydrogels. Finally, results from an *in vivo* mouse study demonstrated that the OHA-DA/PAM/BC hydrogel promoted tissue regeneration and accelerated wound healing. These findings suggested that the OHA-DA/PAM/BC multifunctional hydrogel holds significant potential as a wound dressing and offers an innovative approach to the development of hydrogel-based wound care.

## Supplementary Material

rbaf031_Supplementary_Data
