# Supplementary material for: Bio-inspired lotus-fiber and mussel-based multifunctional hydrogels for wound healing: super-stretchability, self-healing, adhesion and antibacterial properties
Source: Regen Biomater. 2025 Apr 26;12:rbaf031. doi: 10.1093/rb/rbaf031 (PMC12103916; doi:10.1093/rb/rbaf031)
Supplement: rbaf031_Supplementary_Data [file rbaf031_supplementary_data.zip › Supporting Information(4.15).pdf]

Supporting Information

Bio-inspired Lotus-fiber and Mussel-based Multifunctional Hydrogels for Wound Healing: Super-stretchability, Self-healing, Adhesion and Antibacterial Properties

Xiaoling Yang[a.b], Chenchen Li[b], Bo Li[b], Yuanyuan Zhang[b], Jinping Li[b], Na Liu[b], Xin Nie[b], Dawei Zhang[c,*], Ming Zhou[a,*], Xiaoling Liao[b]

[a] School of New Energy and Material, Southwest Petroleum University, Chengdu, 610500, China

[b] Chongqing Engineering Laboratory of Nano/Micro Biomedical Detection Technology, Chongqing University of Science and Technology, Chongqing, 401331, China

[c] Department of Orthopedics, The 960th Hospital of the PLA Joint Logistice Support Force, Jinan, 250031, China.

***Corresponding authors**. Tel.: +86 23 65023257; fax: +86 23 65023706

E-mail address: zdwasy6161@163.com (D.W. Zhang), mr.zhouming@163.com (M. Zhou)

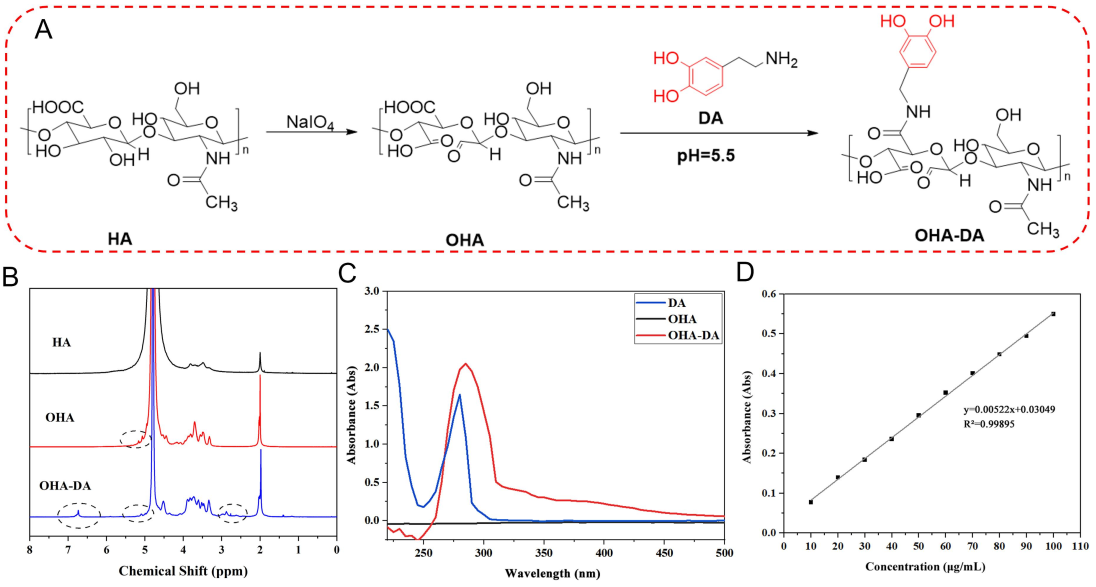

Figure S1. Synthesis and characterization of OHA-DA. Chemical reaction of OHA-DA(A). ^{1}H-NMR spectra of HA, OHA and OHA-DA(B). UV-Vis absorption spectra of DA, OHA and OHA-DA(C). Standard curve of dopamine measured at various concentrations(D).

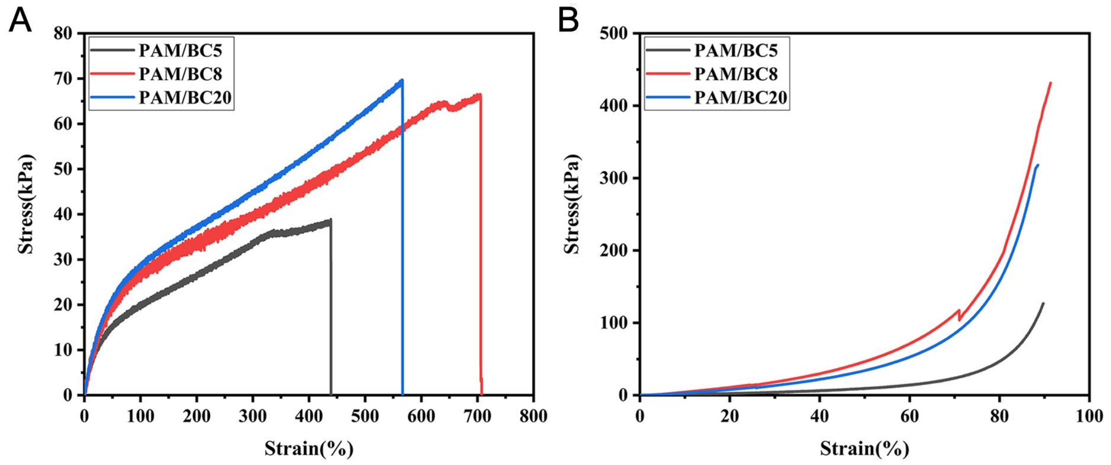

Figure S2. Tensile (A) and compression (B) stress-strain curves of PAM/BC hydrogels with varying BC contents.

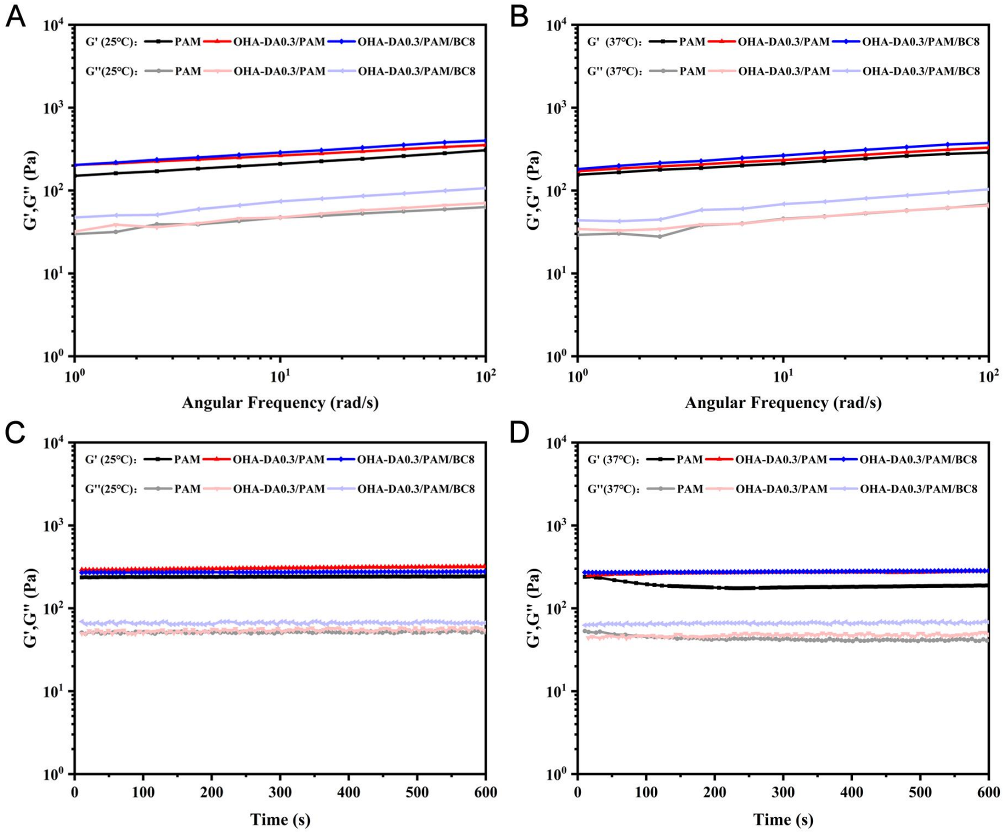

Figure S3. Rheological properties exhibiting the storage modulus G′ and loss modulus G″ of the PAM, OHA-DA0.3/PAM and OHA-DA0.3/PAM/BC8 hydrogels as a function of angular frequency of 1 to 100 rad/s and time sweep in 600s at 25℃ (A, C) and 37℃ (B, D).

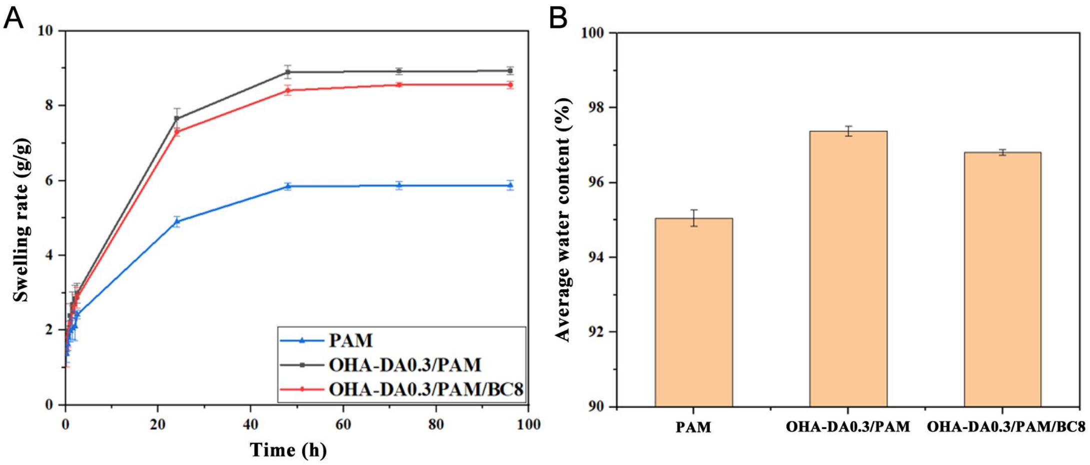

Figure S4. Swelling rate (A) and average water content(B) of PAM, OHA-DA0.3/PAM and OHA-DA0.3/PAM/BC8 hydrogels incubated in distilled water for different times.
